# PDFR and CRY Signaling Converge in a Subset of Clock Neurons to Modulate the Amplitude and Phase of Circadian Behavior in *Drosophila*

**DOI:** 10.1371/journal.pone.0018974

**Published:** 2011-04-29

**Authors:** Seol Hee Im, Weihua Li, Paul H. Taghert

**Affiliations:** Department of Anatomy and Neurobiology, Washington University School of Medicine, St. Louis, Missouri, United States of America; Yale School of Medicine, United States of America

## Abstract

**Background:**

To synchronize their molecular rhythms, circadian pacemaker neurons must input both external and internal timing cues and, therefore, signal integration between sensory information and internal clock status is fundamental to normal circadian physiology.

**Methodology/Principal Findings:**

We demonstrate the specific convergence of clock-derived neuropeptide signaling with that of a deep brain photoreceptor. We report that the neuropeptide PDF receptor and the circadian photoreceptor CRYPTOCROME (CRY) are precisely co-expressed in a subset of pacemakers, and that these pathways together provide a requisite drive for circadian control of daily locomotor rhythms. These convergent signaling pathways influence the phase of rhythm generation, but also its amplitude. In the absence of both pathways, PER rhythms were greatly reduced in only those specific pacemakers that receive convergent inputs and PER levels remained high in the nucleus throughout the day. This suggested a large-scale dis-regulation of the pacemaking machinery. Behavioral rhythms were likewise disrupted: in light∶dark conditions they were aberrant, and under constant dark conditions, they were lost.

**Conclusions/Significance:**

We speculate that the convergence of environmental and clock-derived signals may produce a coincident detection of light, synergistic responses to it, and thus more accurate and more efficient re-setting properties.

## Introduction

The *Drosophila* brain contains ∼150 neurons that display rhythmic expression of clock genes, such as *period* and *timeless*, which act as circadian pacemakers driving diurnal locomotor rhythms. Perturbation of the molecular rhythm in different subsets of clock neurons has been associated with changes to the profile of diurnal behavior [1]–[3], although the system appears to contain substantial plasticity [4], [5]. Molecular oscillators are entrained by external cues like light, and by internal cues, such as the activity of other pacemakers [e.g., 6]. Among photodetectors, the deep-brain photoreceptor CRYPTOCHROME (CRY) is especially critical [7]–[10], and is expressed in many, but not all, clock neurons [10], [11]. The neuropeptide Pigment Dispersing factor (PDF) is a potent internal synchronization signal. It is secreted by ∼10% of pacemakers and it modulates clock phase and/or amplitude in pacemaker-specific fashion [12]–[14].

As they both promote pacemaker synchronization, Cusumano et al. [15] and Zhang et al. [16] asked whether *Pdf* and *cry* also exhibited genetic interactions. Both studies concluded that the pathways do interact and that their combined influence sets the phase of the evening locomotor activity peak and the phase of its underlying molecular oscillation in key pacemakers. In both studies, the behavior and molecular rhythms were retained in double mutant combinations but with altered phases. We also examined these potential interactions, and report firstly that high levels of PDF receptor (PDFR) and CRY are normally co-expressed in precise subsets of clock neurons – pacemaker subsets that support production of the evening activity peak. This finding provides the anatomical basis with which we interpret all other results. With respect to behavior, we find a near-absence of all anticipatory behavior under cycling (light-dark) conditions in flies lacking both pathways, and complete arrhythmicity under constant dark conditions. Finally, we observed that under constant light conditions, partial behavioral rhythms are displayed, but with a phase determined by light intensity. There was a lack of correspondent, clear molecular rhythmicity in LL. Thus our results emphasize: (i) that PDF and CRY signaling pathways normally converge to critically support the amplitude of the circadian molecular oscillator, not just its phase, and (ii) that there is little correlation between pacemaker molecular oscillations and the phase of rhythmic behavior in these specific genetic backgrounds.

## Results

### PDFR-MYC and CRYPTOCHROME are co-expressed in a diverse subset of clock neurons

In a previous study, we reported the expression pattern of PDFR using *pdfr-myc*, a 70 kB transgene from the genomic region that includes *pdfr*, which marks PDFR expression by insertion of a MYC epitope tag at the C-terminus of the receptor [24]. Within the circadian clock network, PDFR is expressed in each of the six major groups of clock neurons, but not in all neurons within each group. In separate studies, the CRYPTOCHROME (CRY) expression pattern was defined with newly-developed antibodies that are highly specific for CRY [10], [11]. Among clock neurons, CRY is also expressed in most groups of clock neurons, but not in all clock neurons. For example, both proteins were reported to be expressed only in three of six LNds and only in six to seven of the ∼17 DN1s [10], [11], [24]. Given that similar numbered subsets of clock neurons were found to express these two signaling molecules, we asked the extent to which CRY and PDFR expression patterns might overlap in the fly brain.

**Figure 1:**
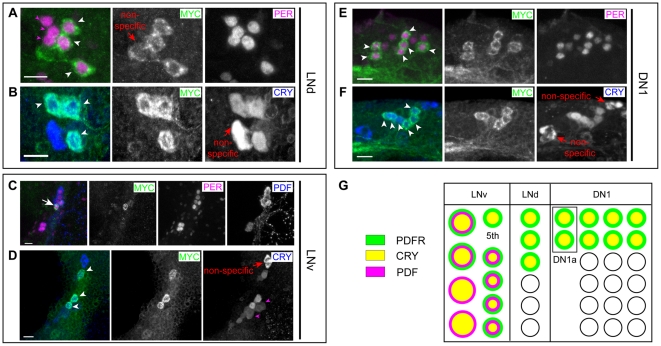
PDFR-MYC and CRYPTOCHROME are precisely co-expressed in the same subsets of clock neurons. PDFR-MYC fly brains were triple-stained with anti-MYC (green), anti-PER (magenta), and anti-PDF (blue) antibodies (A, C, E), or double-stained with anti-MYC (green) and anti-CRY (blue) antibodies (B, D, F). (A) Three LNds of six show strong PDFR-MYC staining (white arrowheads), whereas the others show no PDFR-MYC (magenta arrowheads). (B) Three LNds express both PDFR-MYC and CRY (arrowheads). (C) The 5^th^ s-LNv showed strong staining of PDFR-MYC (arrow). (D) Nine LNv stained with anti-CRY antibody, three of these were also stained with anti-MYC. By reference to results shown in panel C, we assigned the strongest MYC expressing neuron to the 5^th^ s-LNv (arrow). The two remaining MYC(+) neurons are marked with white arrowheads: By size, we speculate these are l-LNv. Two CRY(+) l-LNvs (by size) were not detected with anti-MYC antibody (magenta arrowheads). (E) Six of the 17 DN1s show PDFR-MYC staining at strong levels (white arrowheads), whereas the remaining ones show little or no MYC staining. (F) Six of 15 DN1ps express both PDFR-MYC and CRY (arrowheads). Asterisks (in A, B, D, F) - non-specific staining by either anti-MYC or anti-CRY rabbit antibodies. Scale bars, 10 µm. (G) A summary diagram of the precise PDFR and CRY co-expression in discreet subsets of the three major pacemaker cell groups.

We evaluated PDFR-MYC and CRY expression in pacemaker neurons with antibody labeling, using anti-PER, anti-PDF, and anti-CRY antibodies (Figure 1). As we reported earlier, in the LNd cluster, three of the six neurons express PDFR-MYC (Figure 1A). We found that the three PDFR-MYC(+) LNd neurons were equivalent to the CRY-(+) ones (Figure 1B). In the LNv cluster, the strongest PDFR-MYC stained clock neuron was the 5^th^ s-LNv (Figure 1C). CRY is expressed in all nine cells in the LNv cluster, amongst which CRY showed strong overlapping expression with PDFR-MYC in the 5^th^ s-LNv (Figure 1D). Among the nine LNvs the CRY level is higher in the neurons that are PDFR(+) (Figure 1D). Likewise, in the DN1 cluster (Figure 1E), approximately six to seven of 17 DN1s expressed high levels of PDFR-MYC and as with the LNd group, the PDFR-MYC(+) subset was CRY(+) (Figure 1F). In summary, the highest levels of PDFR expression among pacemaker neurons (and in fact the highest in the entire brain) were matched precisely by high-level CRY expression; we summarize this correspondence in Figure 1G.

### Flies lacking both PDF and CRY signaling display severely-disrupted circadian behavior under constant dark conditions

**Figure 2:**
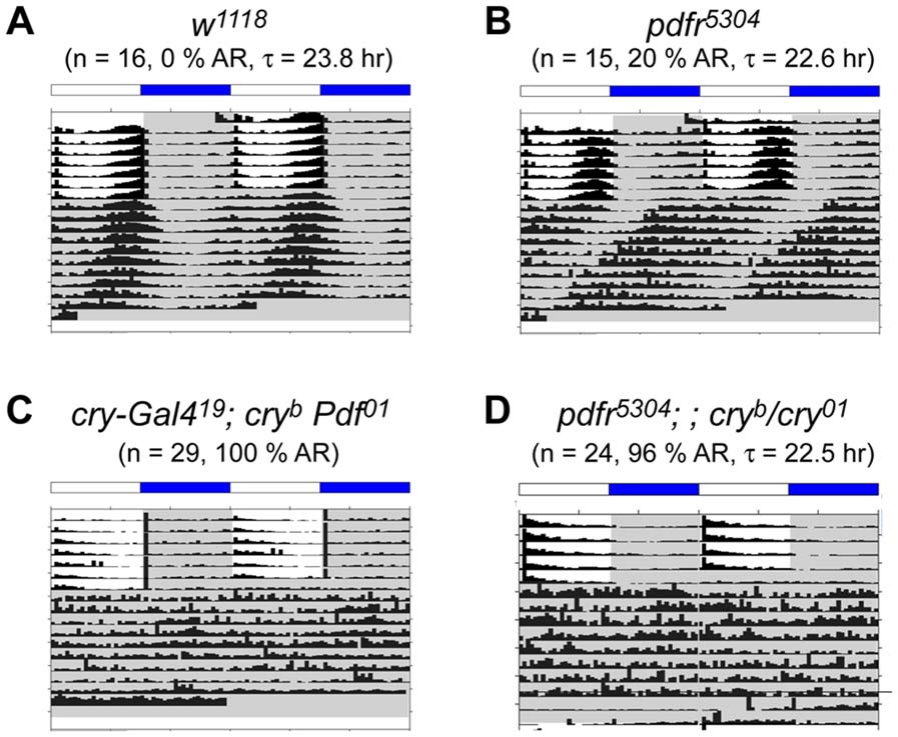
Lack of PDF and CRY signaling causes arrhythmic behavior under DD. Group averaged actograms of each genotype under constant darkness (DD) following LD cycles. (A) *w^1118^* flies; (B) *pdfr^5304^* single mutant flies; (C) *pdfr^5304^; ; cry^b/01^* double mutant flies; (D) *cry-G4^(19)^*; *cry^b^ ss Pdf^01^*double mutant flies. The double mutant flies fail to maintain free running rhythms. For the experiment shown here, the numbers of animals averaged are 16 (A), 15 (B), 29 (C), and 24 (D).

The near-precisely overlapping expression patterns of PDFR and CRY suggested a possible genetic interaction between these two signaling pathways. We hypothesized that, if an interaction between these pathways were critical, loss of both should generate a severe, synthetic phenotype and cause an abnormal circadian behavior. Persistent and robust rhythmic behavior under constant dark conditions is an essential element in the definition of endogenous circadian clock-driven behaviors because such conditions can reveal the fundamental state of the oscillator in the absence of environmental variation. *cry^b^ Pdf^01^* double mutant flies have been previously described by Rouyer and colleagues [15] and *pdfr^5304^; ; cry^b^* double mutant flies by Allada and colleagues [16], but neither of those studies described the behavior of such double mutants in constant dark (DD) conditions. Therefore, to determine the extent of clock-driven rhythmic behavior in the double mutants, we monitored their behavior under DD for up to 9 days (Figure 2, Table 1). Greater than 95% of control flies maintain persistent and robust circadian rhythms, with periods close to 24 hr as shown in Figure 2A and Table 1. For the case of *pdfr^5304^* single mutant flies, about half of the population becomes arrhythmic (50∼70%); the remaining rhythmic flies exhibit short, weakened rhythms (∼22 hr period) (Figure 2B, Table 1; cf., [18]). Previously, Zhang et al. [16] reported on behavior during the first day of DD for *pdfr^5304^; ; cry^b^* flies; they described a modest increase in activity levels around dawn. When we looked at the behavior of *pdfr^5304^; ; cry^b/01^* double and *cry^b^ Pdf^01^* double mutant flies on the first day of DD, we found similar modest levels of anticipatory behavior around dawn - its scale was much less than that of control flies. However, when we extended the study to 9 days in DD, doubly mutant flies displayed near 100% arrhythmicity (Figure 2, Table 1). Thus, in both *pdfr^5304^; ; cry^b/01^* double and *cry^b^ Pdf^01^* double mutant flies, the defective rhythms in behavior were much worse than found in the single *pdfr* or *Pdf* mutants. This suggests that PDF and CRY signaling pathways are together essential for circadian-rhythmic behavior that is normally displayed under constant darkness.

**Table 1:** Summary of behaviors under constant conditions.

Genotypes	Number	AR%	TAU	PWR	WID	SNR
**DD**						
***w^1118^***	37	2%	23.7	65.5	5.4	1.07
***pdfr^5304^***	36	31%	22.7	36.4	4.3	0.63
***w^1118^; ; cry^b^/cry^01^***	76	17%	23.5	58.3	4.8	0.79
***pdfr^5304^; ; cry^b^/cry^01^***	67	86%	23.1	36.7	3.4	0.46
***cry^b^***	20	40%	23.6	47.6	4.5	0.68
***cry-G4 19; cry^b^ Pdf^01^***	60	98%	23.5	15.6	4.0	0.42
***pdfr^5304^; ; [pdfr-myc], cry^b^/cry^01^***	60	29%	23.6	40.4	4.4	0.53
**LL**						
***w^1118^***	62	91%	25.8	24.9	3.2	1.14
***pdfr^5304^***	31	95%	27.5	14.0	1.0	0.19
***w^1118^; ; cry^b^/cry^01^***	59	7%	24.1	75.4	6.3	1.81
***pdfr^5304^; ; cry^b^/cry^01^***	67	60%	22.2	22.3	2.8	0.60
***cry^b^***	43	21%	25.0	54.7	5.2	0.91
***cry-G4 19; cry^b^ Pdf^01^***	46	64%	23.0	28.1	4.2	0.55
***pdfr^5304^; ; [pdfr-myc], cry^b^/cry^01^***	55	16%	24.9	66.1	5.0	1.84

### Flies lacking both PDF and CRY signaling display a severe synthetic phenotype under LD

**Figure 3:**
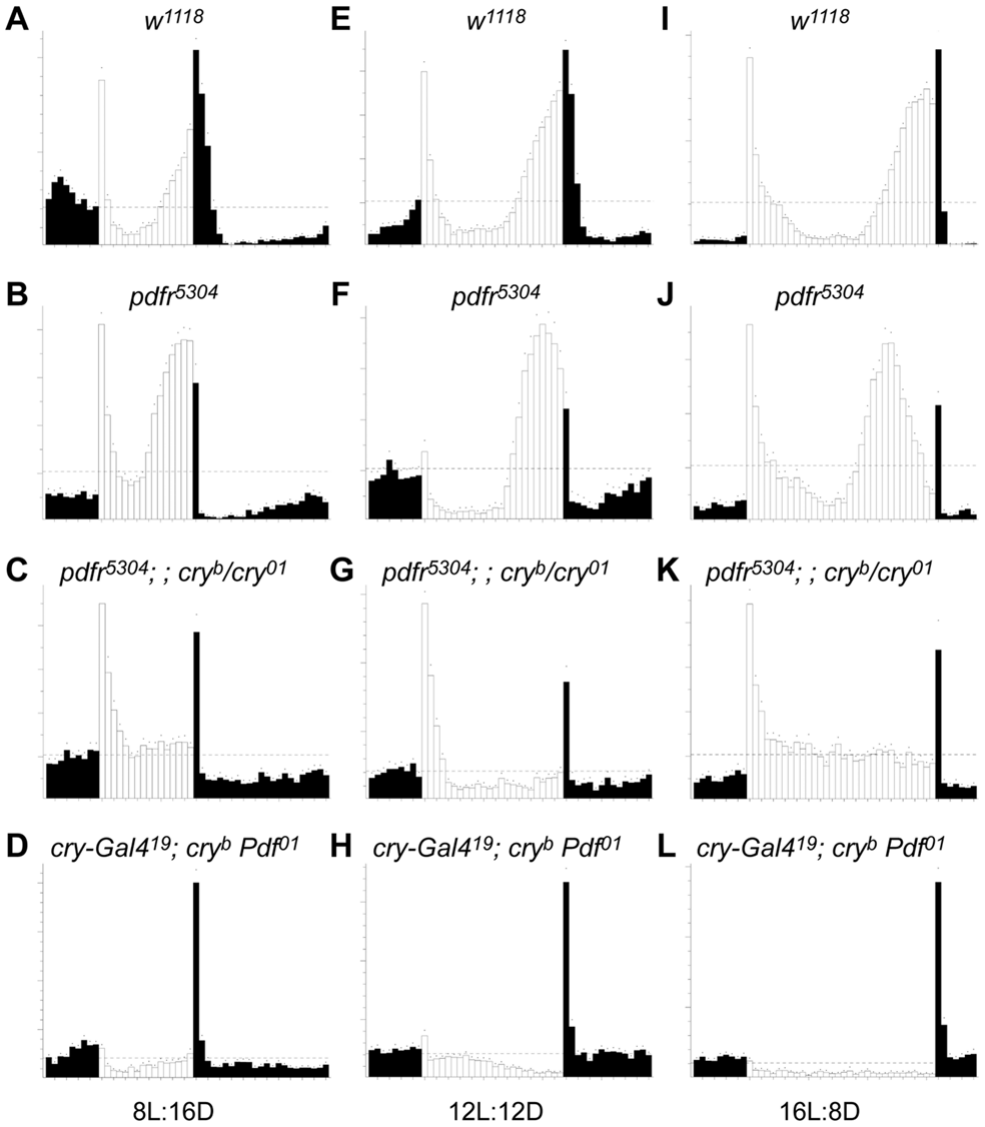
Daily locomotor activities under LD cycles reveal genetic interactions between PDF and CRY signaling pathways. Averaged activity of various genotype flies for a six-day-period under 8∶16 LD (A–D), 12∶12 LD (E–H), and 16∶8 LD (I–L) entrainment conditions. (A, E, I) *w^1118^* control flies; (B, F, J) *pdfr^5304^* single mutant flies; (C, G, K) *pdfr^5304^; ; cry^b/01^* double mutant flies; (D, H, L) *cry-G4^(19)^*; *cry^b^ ss Pdf^01^* double mutant flies. Both double mutant flies display lack of anticipatory peaks under LD cycles. Note that, in *pdfr* single mutants, the longer the day length becomes the more pronounced the advanced evening phenotype. For the experiment shown, the numbers of animals averaged are 32 (A), 31 (B), 32 (C), 32 (D), 30 (E), 14 (F), 15 (G), 32 (H), 32 (I), 31 (J), 31 (K), and 32 (L).

**Figure 4:**
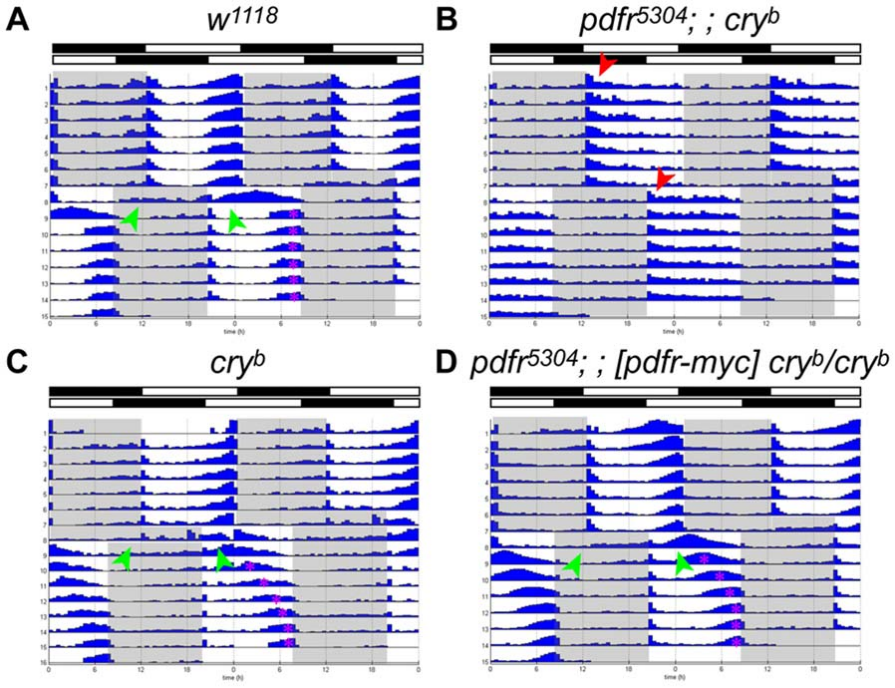
Behavioral responses to changes in light schedules. Flies entrained to a 12∶12 LD cycle were given an eight-hour phase delay. (A) *w^1118^*; (B) *pdfr^5304^; ; cry^b^* double mutants; (C) *cry^b^* single mutants; (D) *pdfr^5304^; ; cry^b^* flies carrying a ∼70 kB *pdfr-myc* transgene. Green arrowheads mark anticipatory behavior of the original light schedule after the delay. Red arrowheads mark the onset of activity by *pdfr^5304^; ; cry^b^* flies following lights-on in the original and delayed schedules. Note that *cry* mutant flies require more cycles to re-entrain to such phase changes [5], [7] and that *pdfr^5304^; ; pdfr-myc*, *cry^b^* (D) displayed a *cry^b^*-like phenotype, indicating rescue of the double mutant behavioral defect. For the experiment shown, the numbers of animals averaged are 16 (A), 16 (B), 16 (C), and 32 (D).

**Figure 5:**
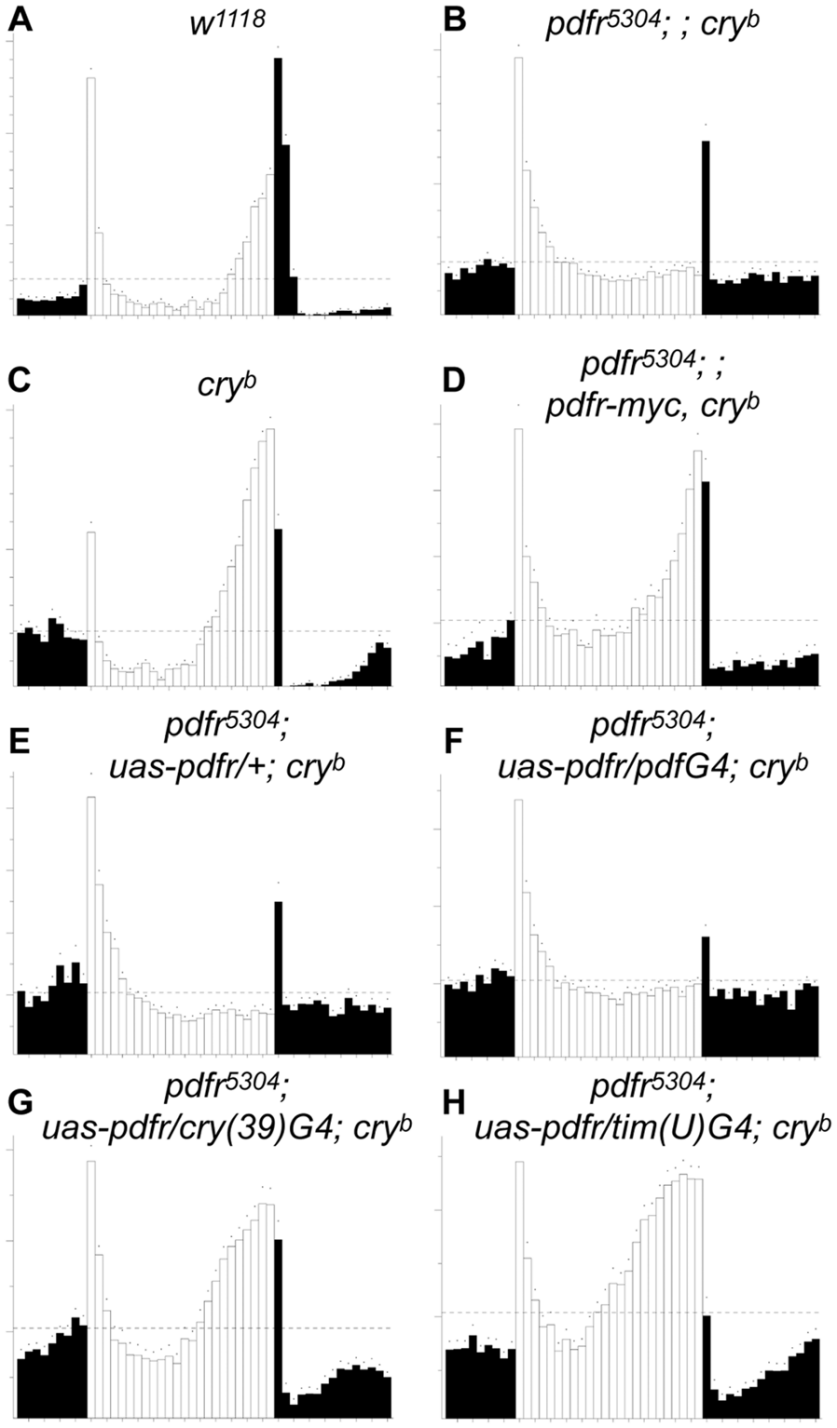
Behavioral defects of *pdfr^5304^; ; cry^b^* double mutants are reversed by restoring normal *pdfr* expression. Flies with either the 70 kB *pdfr-myc* transgene [24] or GAL4-driven *pdfr* expression in the clock network in the *pdfr^5304^; ; cry^b^* double mutant were tested for LD behavior. (A) *w^1118^*; (B) *pdfr^5304^; ; cry^b^* double mutant flies; (C) *cry^b^* mutant flies; (D) *pdfr^5304^; ; pdfr-myc, cry^b^*; (E) *pdfr^5304^; uas-pdfr/+ ; cry^b^* (control flies for GAL4-mediated rescue); (F) *pdfr^5304^; uas-pdfr/Pdf-GAL4 ; cry^b^* (*pdfr* expression restored specifically in PDF cells in the double mutants); (G) *pdfr^5304^; uas-pdfr/cry(39)-GAL4 ; cry^b^* (*pdfr* expression restored specifically in clock cells in the double mutants); (H) *pdfr^5304^; uas-pdfr/tim(uas)-GAL4 ; cry^b^* (*pdfr* expression restored in clock neurons and many other brain cells in the double mutants). For the experiment shown, the numbers of animals averaged are 32 (A), 31 (B), 29 (C), 28 (D), 21 (E), 32 (F), 20 (G), and 31 (H).

With respect to behavior under light∶dark cycling conditions (12 hr light∶12 hr dark – 12∶12 LD), we again found a severe circadian phenotype in the double mutant flies. In LD, control flies (*w^1118^* or a single mutant *pdfr^5304^*) display anticipatory peaks before the lights-on and/or light-off (Figure 3E, 3F). However, for the case of the *pdfr cry* double mutant (*pdfr^5304^; ; cry^b/01^*), we observed several defects (Figure 3G), similar to what was reported by Zhang et al. [16]. (1) Evening anticipatory peaks were greatly attenuated. We also noticed an additional defect, not previously emphasized: (2) Following a pronounced bout of activity after lights-on, activity levels decreased linearly for two hours, and remained very low until the lights-off time. Here we present the behavior of double mutant flies that are trans-heterozygous for *cry* allele (*pdfr^5304^; ; cry^b/01^*), but we also tested double mutants that were homozygous for the different *cry* alleles (*pdfr^5304^; ; cry^b^* and *pdfr^5304^; ; cry^01^*) similar to those studied by Zhang et al. [16]: and the results were comparable (Figure 4B, Figure 5B, Data not shown). The interaction of *pdfr* with *cry* reflected activation of the receptor by the neuropeptide PDF, as the behavioral defects of *cry^b^ Pdf^01^* flies were similar; no anticipation of LD transition and a pronounced startle effect in response to lights-off (Figure 3H; cf. [15]). We observed additional defective features in the behavioral syndrome of *cry^b^ Pdf^01^* double mutant flies. These flies exhibit (3) a smaller startle response to the light-on signal, than do *pdfr^5304^; ; cry^b/01^* double mutants, and much larger lights-off startle response. Finally, they display (4) an apparent negative masking effect to light compared to the single mutant control lines and compared to *pdfr^5304^; ; cry^b/01^* double mutants. These additional defective features (3 and 4) were not observed by Cusumano et al. [15].

The essential features of the diurnal activity profiles were not a function of the specific lighting cycles as shown by testing the same mutant combinations under different LD cycles. In both short day (8∶16 LD) and long day (16∶8 LD) conditions, we found the same defects as shown in 12∶12 cycles (Figure 3C, 3D, 3K, 3L). Overall, rhythmic behavior of double mutant flies was dramatically suppressed in all three LD conditions tested.

As mentioned above, elevated activity levels immediately after the lights-on was pronounced in *pdfr^5304^; ; cry^b/01^* double mutants. This elevated activity could represent endogenous clock driven activity as previously suggested [16], or positive masking behavior, or a mixture of both. To discriminate between these possibilities, we employed a phase-shifting experimental design. When we applied an eight-hour phase delay to the lights-on signal in a 12∶12 LD cycle, control flies showed anticipatory activity in the first cycle after the shift that corresponded to the original light-dark regimen (green arrowheads, Figure 4A). However, the *pdfr^5304^; ; cry^b^* double mutants showed little evidence of such anticipation to the original light-dark regimen. Notably, *pdfr^5304^; ; cry^b^* exhibited activity level increases immediately in response to the new lights-on signal (Figure 4B). Thus the “morning” activity of these double mutant flies exhibits little evidence of gating. This observation supports the hypothesis that their elevated activity immediately after the lights-on signal reflects a preponderance of masking behavior overlaying a minor, clock-driven component.

### *pdfr; ; cry* behavioral defects can be rescued by specific restoration of *pdfr* expression to clock neurons

To confirm that the phenotype of the double mutant is due in part to loss of PDF signaling, we sought to rescue the behavioral defect by restoring PDFR expression in the *pdfr^5304^; ; cry^b^* double mutant flies. First we used the 70 kB *pdfr-myc* transgene, which can rescue all the circadian locomoter defects of *pdfr^5304^* single mutant [24]. As expected, the 70 kB *pdfr-myc* transgene can rescue *pdfr^5304^; ; cry^b^*: the rescued genotype now behaves just like *cry^b^* single mutant following an eight hour phase delay (Figure 4C, 4D), and exhibits normal behavior under LD cycles (Figure 5D). As previously described by Cusomano et al. [15], we also utilized GAL4-driven *pdfr* restoration to specifically ask where in the clock network, PDF receptor expression is required. GAL4 lines that were tested include *Pdf*-GAL4 (Figure 5F), to restore PDFR expression only in the PDF(+) cells [17], *cry(39)*-GAL4 (Figure 5G), to generally restore the receptor expression in the clock network [25], and *tim(uas)*-GAL4 (Figure 5H), to cover even wider brain areas [26]. As expected, *Pdf*-GAL4 failed to restore the lost evening peak of the double mutants, whereas *cry(39)*-GAL4 and *tim(uas)*-GAL4 successfully rescued the evening peak, suggesting that, for these functions under study, the receptor is normally required in non-PDF-expressing clock neurons that co-express high levels of PDFR and CRY.

### PER cycling is attenuated within a subset of clock pacemakers in flies that lack both PDF and CRY signaling

**Figure 6:**
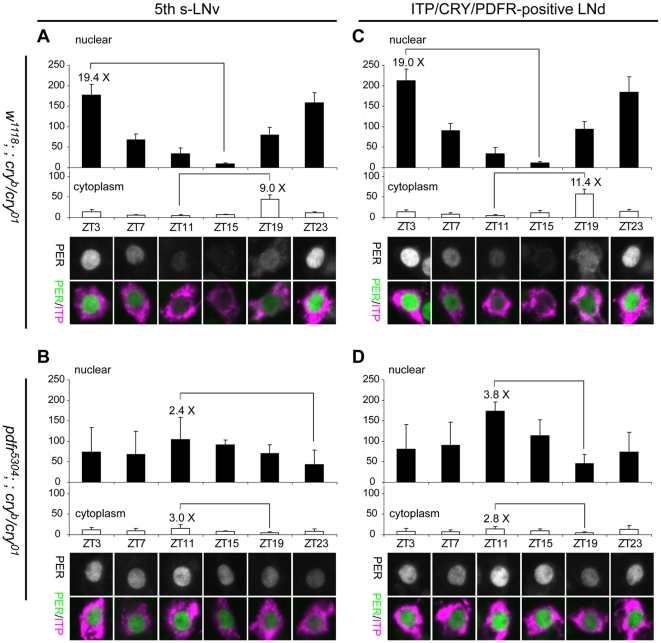
LD Molecular rhythms in the 5^th^ s-LNv and LNd are deranged in the double mutants. At various time-points, PER levels were monitored in the nucleus (filled histograms) and cytoplasm (open histograms) of the 5^th^ s-LNv (A, B) and the ITP(+) LNd (C, D). (A) In the 5^th^ s-LNv of *w^1118^; ; cry^b/01^*, PER levels in the nucleus and cytoplasm are robustly cycling: nuclear amplitude rhythm – 19.4-fold; cytoplasmic amplitude rhythm – 9.0-fold. ANOVA test revealed that the differences in nuclear staining levels are significant (P<0.0001). (B) In the 5^th^ s-LNv of *pdfr^5304^; ; cry^b/01^*, PER staining is always found in the nucleus with very low amplitude rhythms and no phase difference between nucleus and cytoplasm: nuclear amplitude rhythm –2.4-fold; cytoplasm amplitude rhythm – 3.0-fold. ANOVA test revealed that the difference in this group is significant (P = 0.03). (C) In the ITP(+) LNd of *w^1118^; ; cry^b/01^*, PER levels in the nucleus and cytoplasm are robustly cycling, nuclear amplitude rhythm – 19.0-fold; cytoplasmic amplitude rhythm – 11.4-fold. ANOVA test revealed that the difference in this group is significant (P<0.0001). (D) In the ITP(+) LNd of *pdfr^5304^; ; cry^b/01^*, PER staining is always found in the nucleus with very low amplitude rhythms and no phase difference between nucleus and cytoplasm: nuclear amplitude rhythm – 3.8-fold; cytoplasmic amplitude rhythm – 2.8-fold. ANOVA test revealed that the difference in this group is significant (P<0.0001). Results from post-hoc statistical tests are presented in Table 2.

To ask if the behavioral defects that result from loss of both PDF and CRY signaling are correlated with malfunctions of the molecular clock in pacemaker neurons, we monitored PER cycling at six time points under LD cycles (Zeitgeber Time 3, 7, 11, 15, 19, and 23; ZT0 refers to the time of lights-on - Figure 6). We compared the double mutant flies (*pdfr^5304^; ; cry^b/01^*) to *cry* single mutant flies (*w^1118^; ; cry^b/01^*). We first examined the molecular rhythms in those clock neurons that normally co-express PDFR and CRY. We used neuropeptide ITP co-staining as a marker to identify those specific neurons, and also as a counterstain for PER's subcellular localization. ITP is expressed in the 5^th^ s-LNv and in one of the PDFR/CRY(+) LNds [23].

**Table 2:** Post-hoc tests for results illustrated in Figures 6 and 10.

Figure	Genotype	Neuron	P<0.001	P<0.01	P<0.05
6A	*w^1118^; ; cry^b/01^*	5^th^ s-LNv	ZT3 vs ZT7, 11, 15, 19	ZT11 vs ZT19	ZT11 vs ZT15
			ZT7 vs ZT15, 23		
			ZT11 vs ZT23		
			ZT15 vs ZT19, 23		
			ZT19 vs ZT23		
6B	*pdfr^5304^; ; cry^b/01^*	5^th^ s-LNv			ZT11 vs ZT23
6C	*w^1118^; ; cry^b/01^*	ITP(+) LNd	ZT3 vs ZT7, 11, 15, 19		
			ZT7 vs ZT11, 15, 23		
			ZT11 vs ZT19, 23		
			ZT15 vs ZT19, 23		
			ZT19 vs ZT23		
6D	*pdfr^5304^; ; cry^b/01^*	ITP(+) LNd	ZT3 vs ZT11	ZT15 vs ZT19	ZT11 vs ZT15
			ZT7 vs ZT11		
			ZT11 vs ZT19, 23		
10B	*pdfr^5304^; ; cry^b/01^*	5^th^ s-LNv	CT65 vs CT77	CT71 vs CT77	
10C	*pdfr^5304^; ; cry^b/01^*	ITP(+) LNd	CT65 vs CT77	CT77 vs CT83	
10D	*pdfr^5304^; ; cry^b/01^*	PER(+) DN1		CT65 vs CT71	CT71 vs CT77

Tukey-Kramer multiple comparisons post-hoc test results.

**Figure 7:**
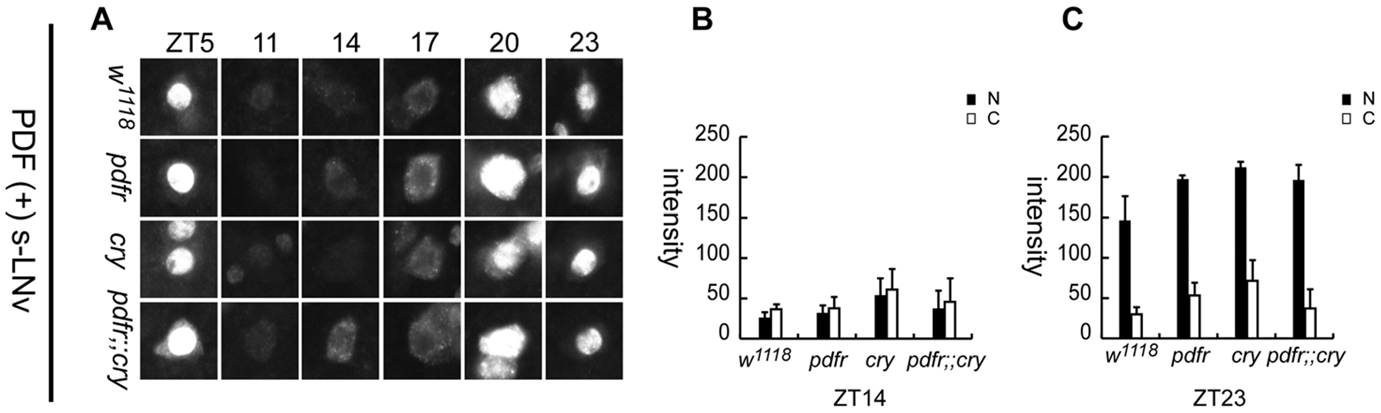
Quantification of PER intensity in the nucleus and cytoplasm of PDF(+) s-LNvs in LD cycles. (A) PER staining in single focal plane images of the PDF(+) s-LNvs at various time points. s-LNvs were chosen by size and PDH immunoreactivity. All four genotypes of flies show normal cycling of PER in the s-LNvs. (B and C) Quantifications of the mean pixel intensities of PER in the nucleus (filled histograms) and cytoplasm (open histograms) at ZT14 (B) and ZT23 (C) (n = 5∼6).

In the control, the 5^th^ s-LNv and the ITP-(+) LNd showed a normal progression of PER protein rhythms, both in intensity and sub-cellular localization (Figure 6A, 6C). The amplitude of PER intensity rhythm (which was calculated by ratio of mean intensity for the highest to the lowest) was 19.4 for the nuclear domain and 9.0 for the cytoplasmic domain of the 5^th^ s-LNv, whereas for the ITP-(+) LNd, the ratios were 19.0 for the nuclear domain and 11.4 for the cytoplasmic domain. In the double mutant flies, we found several abnormalities in PER cycling in these same pacemakers. First, there is an altered phase in PER staining intensity measured in the nuclear domain, with the peak at ZT11 for both the 5^th^ s-LNv and ITP-(+) LNd (Figure 6B, 6D). This result is in basic agreement with that reported by Cusumano et al. [15] and by Zhang et al. [16], where both groups monitored whole cell PER intensity. Cusumano et al. [15] and Zhang et al. [16] both referred to the defect as an “antiphasic” PER rhythm which could potentially explain the behaviors they reported. However, we report three additional defects to the molecular rhythms in these critical pacemaker types, not previously reported. (i) PER amplitude is greatly reduced: the amplitude of PER intensity of the 5^th^ s-LNv was just 2.4 and that of the ITP(+) LNd was 3.8 – that represents a loss in rhythm amplitude of up to ∼10 fold (Figure 6B, 6D). (ii) A second important change in the double mutants is that the subcellular localization of PER does not change: the PER proteins resided mainly in the nucleus and there was only a very slight amount of PER detected in the cytoplasm of either pacemaker type (Figure 6B, 6D). (iii) Finally, unlike the oscillations in control genotypes, which display a 6–8 hour phase difference between peak nuclear versus peak cytoplasmic PER intensity levels, the double mutants have coincident nuclear and cytoplasmic peaks at ZT11. These combined features argue strongly that molecular rhythms in these neurons of the double mutant flies are greatly attenuated and barely functioning. As was reported earlier [15], [16], we found that the PDF(+) s-LNvs showed very normal PER cycling in the double mutants (Figure 7). The latter result suggests the effects on PER rhythm produced by loss of these two signaling systems are confined to those pacemaker that normally have high level expression of PDFR and CRY.

Thus, the strong behavioral disruptions exhibited by the double mutant flies were strikingly matched by a particular syndrome of molecular disturbances to a subset of critical pacemaker neurons. The most pronounced disturbance was the severe attenuation of the PER molecular rhythm, and was limited to a subset of LNd and to the 5^th^ small LNv, both of which express high levels of PDF receptor and CRY.

### Flies lacking PDF and CRY signaling display partial rhythmic behaviors under constant light conditions

**Figure 8:**
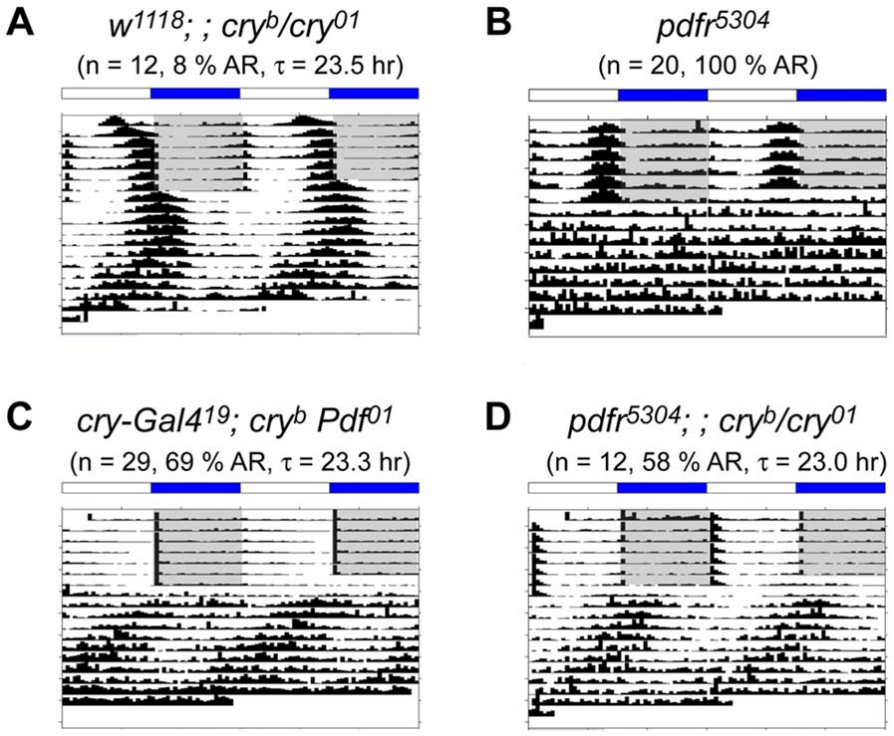
Lack of PDF and CRY signaling causes weak, short behavioral rhythms under LL. Group-averaged actograms of each genotype. (A) *w^1118^; ; cry^b/01^* single mutant flies; (B) *pdfr^5304^* single mutants; (C) *pdfr^5304^; ; cry^b/01^* double mutant flies; (D) *cry-G4^(19)^*; *cry^b^ ss Pdf^01^* double mutant flies.

Expression of rhythmic behavior by mutant flies under constant light conditions is routinely used to detect defects in light perception. For example, mutations of the gene encoding the CRY photoreceptor renders flies rhythmic in LL, in contrast to control flies which normally become arrhythmic [27]. In the case of flies doubly-mutant for PDF and CRY signaling pathways, their loss of strong rhythmic behavior in LD conditions suggests that these double mutant flies have lost functional clock and/or light entrainment. To the contrast to what we expected, the behavioral defects of the double mutants (e.g., *cry^b^ Pdf^01^* or *pdfr^5304^; ; cry^b/01^*) under constant light (LL) were different from complete arrhythmicity observed under DD conditions. Control flies (*w^1118^*) and *pdfr^5304^* single mutants are arrhythmic under LL (data not shown, Figure 8B), while *cry* single mutants show robust rhythmic behavior under LL, as previously described (Figure 8A; cf. [27]). The combination of *pdfr* and *cry* (*pdfr^5304^; ; cry^b/01^*) or *Pdf* and *cry* (*cry^b^ Pdf^01^*) resulted in partial rhythmic behavior, with about 40% of flies showing rhythmicity (Figure 8C, 8D, Table 1). These rhythmic flies have a slightly shorter period (∼23 hr) and a low power, similar to *Pdf* or *pdfr* single mutants under DD conditions. Moreover, the LL rhythmicity is not maintained for long, in that after four to five cycles overall amplitude of rhythm is greatly attenuated. Cusumano et al. [15] also reported similar rhythmic behavior of *cry^b^ Pdf^01^* double mutant flies under LL conditions. We found one significant difference from their study [15] in the phasing of LL behavior. That is potentially significant because it bears on the state of the oscillator in flies lacking both signaling pathways. Cusumano et al. [15] found a peak phase of ZT1.6, consistent with a phase shift of the oscillator driving the locomoter activity rhythm from the evening to the morning. In our observations, however, the LL rhythmic behavior originated from an evening phase of LD activity (ZT11.98). We found this evening phase behavior in both *pdfr^5304^; ; cry^b/01^* double (ZT13.53) and *cry^b^ Pdf^01^* double mutant flies.

**Figure 9:**
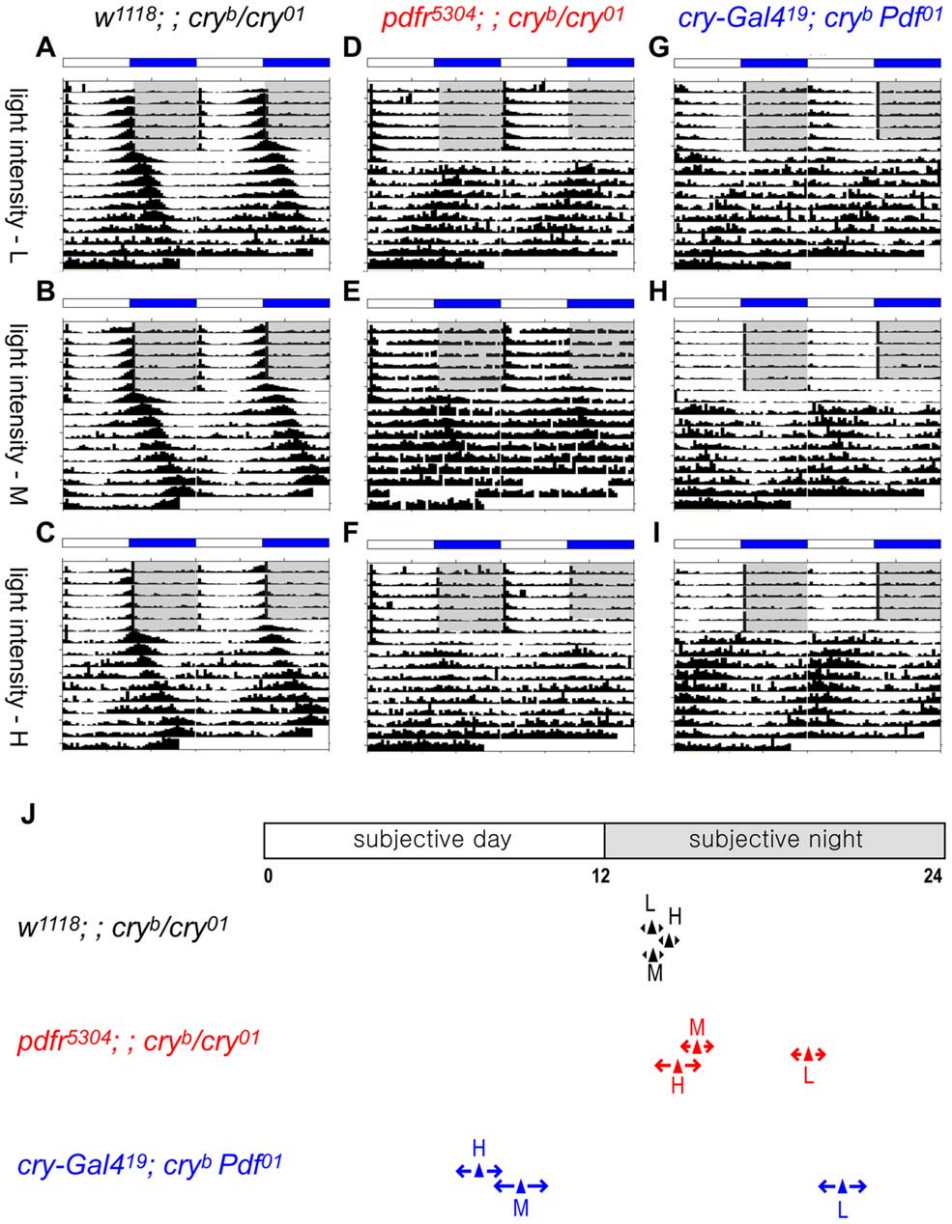
Light intensity affects the phase of LL rhythmic behavior of the double mutants. (A–I) Group-averaged actograms of each genotype. Low light intensity – top row. Middle light intensity – middle row. High light intensity – bottom row. (A–C) *w^1118^; ; cry^b/01^* flies; (D–F) *pdfr^5304^; ; cry^b/01^* flies; (G–I) *cry^b^* flies. (J) Averaged phase markers under different light intensity LL conditions; arrow widths display standard errors of the populations. Statistical analyses were performed between behavioral peak phases under different light intensity conditions of the same genotype flies. ANOVA test for both double mutant flies showed that the difference was significant (P<0.0001). Tukey-Kramer multiple comparisons post-hoc test results revealed P<0.0001 for [L vs M] and for [L vs H] for both double mutant genotypes. For the experiment shown, the numbers of animals averaged are 27 (A), 30 (B), 32 (C), 30 (D), 32 (E), 30 (F), 30 (G), 32 (H), and 32 (I).

To potentially resolve these conflicting observations, we determined if different light intensities might contribute to phase differences in LL activity rhythms. We surveyed three different light intensity conditions, including light intensity conditions similar to those reported by Cusumano et al. [16] (see Materials and Methods), and saw clear evidence that the phase of LL rhythmic behavior changes in response to different light intensities. For *pdfr^5304^; ; cry^b/01^* double mutants, the phase at low light intensity LL conditions corresponded to late subjective night (ZT19.21), whereas the phases at medium to high light intensity conditions were at ZT15.35 and ZT14.56, respectively (Figure 9). For *cry^b^ Pdf^01^* double mutants, the phase at low light intensity conditions was at ZT20.40, whereas at medium and high light intensity conditions they moved to the late subjective day, ZT9.13 and ZT7.59 (Figure 9). Interestingly, this light intensity-dependent phase change only occurred in the flies that lost both PDF and CRY signaling. In *cry* single mutants (*w^1118^; ; cry^b/01^*), regardless of light intensity, the evening phase remained near the light-off time point of the previous entrainment period, ZT13.65 (L), ZT13.72 (M), and ZT14.28 (H).

**Figure 10:**
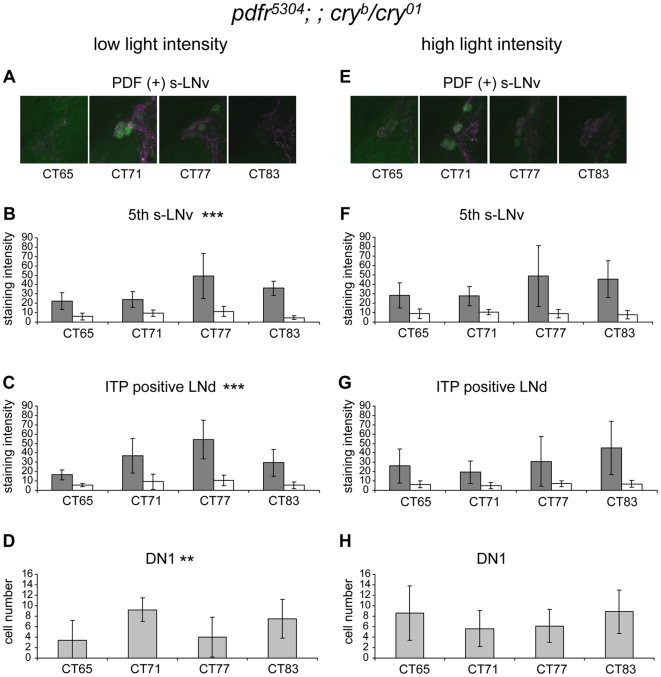
PER molecular rhythms in double mutants under different light intensity LL conditions. (A–D) low intensity; (E–H) high intensity. (A, E) PDF(+) s-LNv; (B, F) 5^th^ s-LNv; (C, G) ITP(+) LNd; (D, H) DN1. Filled histograms - nuclear values and open histograms - cytoplasmic values (B, C, F, G). (A, E) PDF(+) s-LNv (magenta) showed robust PER (green) staining rhythms under both light intensity conditions. (B–D, F–H) For the other cells examined, a statistically-significant amplitude rhythm was shown under low light conditions for the 5^th^ s-LNv and for the ITP(+) LNd. None of the cells showed a significant amplitude rhythm under high light intensity. (B) At low intensity, both nuclear and cytoplasmic peaks in the 5^th^ s-LNv occurred at CT77: nuclear amplitude rhythm – 2.2-fold; cytoplasmic amplitude rhythm – 2.5-fold. ANOVA test revealed that the difference in this group is significant (P<0.0003). (C) In the ITP(+) LNd, both nuclear and cytoplasmic peaks occurred at CT77: nuclear amplitude rhythm – 3.3-fold; cytoplasmic amplitude rhythm – 2.0-fold. ANOVA test revealed the group difference is significant (P<0.0001). (D) PER(+) DN1 neurons were counted at four time points under low light intensity conditions. The rhythm in PER(+) DN1 didn't show 24 hour rhythms. ANOVA test P value was 0.0038. (F) At high intensity, nuclear peak in the 5^th^ s-LNv occurred at CT77 and cytoplasmic peak occurred at CT71: nuclear amplitude rhythm – 1.8-fold; cytoplasmic amplitude rhythm – 1.4-fold. ANOVA test indicated the group difference was not significant (P = 0.0527). (G) In the ITP(+) LNd, the nuclear peak occurred at CT83 and the cytoplasmic peak at CT77: nuclear amplitude rhythm – 2.3-fold; cytoplasmic amplitude rhythm – 1.4-fold. ANOVA test revealed that the difference in this group is not significant (P = 0.08). (H) PER(+) DN1 neurons were counted at four time points under high intensity conditions. The rhythm in PER(+) DN1 showed a peak at CT65, but it was not statistically significant. Results from post-hoc statistical tests are presented in Table 2.

To define potential cellular sources of the behavioral rhythmicity shown in LL with different light intensity conditions, we immunostained fly brains on LL Day 3 and looked for evidence of restored PER rhythmicity. When examined at four times points spanning from CT65 to CT83, most neurons displayed a molecular rhythm phenotype very similar to what we previously observed under LD cycles. Namely, PER rhythms in the 5^th^ s-LNv and ITP(+) LNd were low in amplitude (Figure 10B, 10C, 10F, 10G), while those of PDF(+) s-LNvs were robustly cycling (Figure 10A, 10E). Specifically, under low light intensity conditions, the 5^th^ s-LNv and ITP(+) LNd showed a low amplitude in PER staining intensity with a peak around CT77 (Figure 10B, 10C). At high light intensity conditions, 5^th^ s-LNv showed a peak at CT77 whereas the ITP(+) LNd showed a peak at CT83 (Figure 10F, 10G). We also surveyed DN1 as well, in which PER(+) neurons were counted, and we found again no obvious rhythms in either light conditions (Figure 10D, 10H). The PDF(+) s-LNvs showed robust high amplitude cycles of PER staining intensity and sub-cellular distribution in both light conditions, with a peak at CT71, similar to those shown in LD cycles (Figure 10A, 10E).

## Discussion

Our anatomical data pinpoints specific *Drosophila* pacemakers, among its ∼150 prominent circadian oscillators, as sites of high-level CRY and PDFR expression. This precise co-localization of two signaling systems within the circadian neural circuit supports a hypothesis that CRY and PDF play convergent roles in shaping diurnal rhythmic outputs. Previous studies of flies mutant for both the PDF and CRY signaling systems concluded that their physiological significance for the clock system lies in their ability to delay the evening activity peak away from a default morning phase [15], [16]. Our studies lead us to a substantially different conclusion: that the CRY and PDF signaling are essential elements to sustain PER oscillations in key pacemakers, not just to delay their phases but to maintain their proper amplitudes. Hence, they are critical elements in circadian entrainment because together they promote the normal phase and amplitude of behavioral rhythms.

The behavior of the double mutant flies in LD is not easily explained. Activity appears suppressed in the dark phase, followed by increased activity with lights-on that gradually diminishes until lights off. Zhang et al. [16] argued that the predominant bout of activity that occurs after the lights-on signal represents the normal evening peak now phase-advanced by ∼12 hr. They showed that it was still present (albeit, highly reduced) for a single day in DD (DD1). Cusumano et al. [15] did not specifically refer to the morning activity in LD as a phase-advanced evening peak. We propose a different explanation for this same LD behavior. Namely, that there is virtually no clock-driven behavior in LD cycles in double mutant flies, due to severely compromised clock functions in their critical pacemakers. Instead the activity bout appearing with lights-on reflects a direct response to light, as supported by two behavioral observations. (1) In each of three different LD cycles, elevated locomotor activity was always in smooth register with the prevailing light-on conditions. (2) In phase-delaying light conditions, double mutant stocks show little if any anticipation for the phase of the original lights-on signal. Instead, they display a strong upswing of activity at the new lights-on signal. Hence the LD activity rhythm of double mutant flies shows little evidence of gating by an internal clock, but instead represents primarily a masking response. This conclusion is confirmed by the complete arrhythmicity these flies show under DD conditions (a hallmark test for the presence of a durable circadian oscillator).

The effects of the double mutant combinations on the molecular oscillator are profound and remarkably cell-specific. Oscillations of PER staining intensity in small LNv appear normal but are heavily affected in the 5^th^ small LNv and the ITP+-LNd. Both Cusumano et al. [15] and Zhang et al. [16] measured PER staining intensity oscillations in the latter cells, and reported normal cycling but with a dramatic change in phase. We measured PER staining intensities - and sub-cellular distributions - and conclude the molecular oscillator in those pacemakers is not simply phase-altered, but instead severely deranged. Staining intensities show a slight rhythm with a phase-advanced peak [cf.15], [16]. However, (i) the amplitude of the PER oscillation is (up to 10-fold) diminished, (ii) considerable PER levels persist in the nucleus throughout the day (with presumably consequent, continuous transcriptional repression), and (iii) the normal ∼8 hr phase difference between cytoplasmic and nuclear PER accumulations (that is critical for normal PER timing functions (e.g., Curtin et al., 1995) is completely lost. While the slight peak of PER staining intensity indicates some continued flux in PER levels, all other measures argue that these specific pacemakers are poorly functioning, as exemplified by their inability to effectively degrade PER. Thus lack of PDF and CRY signaling does not merely affect the phase of the molecular oscillation in certain pacemakers. Instead, they are also required to sustain molecular oscillation, even under strong, cycling LD conditions. Removing CRY from either a *Pdf* or *pdfr* mutant stocks dispels all DD constant condition rhythmicity, and thus reveals a previously unsuspected, light-independent CRY function that helps sustain molecular rhythms.

In spite of their poor rhythmic behavior in LD and DD, these double mutant flies display reasonably strong rhythmic behavior in LL. Its features include a shorter period and ∼50% arrhythmicity among populations, which resemble the behavioral syndrome of flies lacking *Pdf* or *pdfr* in DD [13], [17], [18]. Hence it is reasonable to suppose that whichever pacemakers drive this behavior in LL, their formal properties (perhaps even their identities) equal those for DD behavior. Cusumano et al. [15] argued that this rhythmic behavior displayed an abnormal “morning” phase and hence was correlated with the ani-phasic PER molecular rhythms they had noted in LD in the 5^th^ small LNv and CRY+ LNd. These features led them further to suggest that the fundamental role of PDF and CRY is to delay the evening peak away from a default morning phase. We contend this conclusion is incomplete: we found that the phase of LL rhythmic behavior is not locked to the subjective morning, but can move substantially, as a function of light intensity. Furthermore, behavioral phase is not correlated to the molecular rhythm phase of either the 5^th^ small LNv or ITP(+)-LNd pacemaker. Finally, the PER rhythms that can be measured in these pacemakers in different LL conditions reflect heavily deranged molecular oscillators, as noted above. Hence, we do not consider the roles of PDF and CRY signaling to merely support an obligate delay of the evening peak, away from a morning phase. Instead we favor the hypothesis that together PDF and CRY signaling are instrumental to set the phase and the amplitude of the evening peak.

We assume that PDF signaling, like CRY signaling [8], [10], [29], [30], is phasic, and that it varies over the course of the day [cf. 31]. If so, these data indicate that circadian oscillator amplitude is likewise regulated by these two inputs on a daily basis. Vitaterna et al. [32] previously showed that regulation of circadian amplitude affects the strength of entrainment: *Clock* mutant mice display strong (phase 1) re-setting properties, which likely derive from lowered circadian amplitude. Therefore, by regulating circadian rhythm amplitude, convergent PDF and CRY could support optimal pacemaker entrainment in *Drosophila* at critical nodes in the circadian neural circuitry.

The CRY and PDF (or PDFR) pathways both reflect light inputs, but they are not simply redundant as their behavioral phenotypes are very distinct. While CRY input is a direct read-out of light levels, the PDF input partly reflects the same light information that is filtered through synaptic relay from retinal and H-B eyelet photoreceptors, and through the durable clock machinery of the LNv pacemakers. The benefits of such a comparative direct/indirect calculation may involve accuracy, efficiency, or both. First, entrainment based on comparative inputs may promote more accurate timekeeping. Foraging desert ants return home by reliance on an internal path integrator [33]. However that mechanism is prone to errors and is in part corrected by information about the immediate environment in the form of cartographic images. This direct input of environment information is placed onto a grid work representation derived from the path integrator [34]. Ant navigational accuracy thus depends (in part) on a convergence of internal computations and environmental inputs. A second model to explain the productive convergence of PDF and CRY pathways invokes signaling synergism [35]. The spatial convergence of PDF and CRY signaling at critical pacemaker nodes may create a coincidence detector, whose super-additive read-out(s) could alter clock properties more efficiently than either one alone.

Finally, Cusumano et al. [15] argued that the role of PDF in shaping diurnal behavior was clock-independent for two reasons. First, because the double mutant phenotype was rescued when PDF rescue was restricted to large LNv (which contain quickly-damping molecular oscillators), and second, because flies lacking a functional clock in LNv under red light cycling condition did not phenocopy the behavior of the double mutant (*cry^b^ Pdf^01^*). We consider that conclusion premature. Firstly, the demonstration that PDF in large LNv alone is sufficient for rescue is a far cry from showing that PDF in large LNv is necessary, or (equally important) that PDF expressed in small LNv is neither necessary nor sufficient. Hence this critical PDF could well be derived from the durable (small LNv) clock source. Secondly, use of red light to simulate a *cry* mutation in this experiment is an untested proposition. Hence this design is not an ideal one with which to test the hypothesis. Clearly, the field could use direct observations on the potential role of the clock in shaping PDF release, and how such phasic signaling might converge at specific nodes of the circadian circuitry to complement and perhaps synergize with CRY-mediated light inputs.

## Materials and Methods

### Fly strains

*pdfr^5304^*, *cry^b^*, *cry^01^*, *Pdf^01^* were previously described [7], [9], [17], [18]. For double mutants, we crossed *pdfr^5304^; ; cry^b^ ss* and *pdfr^5304^; ; cry^01^* to acquire trans-heterozyote double mutant flies (*pdfr^5304^; ; cry^b/01^*). We acquired *cry-GAL4^19^; cry^b^, Pdf^01^* flies from F. Rouyer, and this fly strain was previously described [15], and in the Results and Discussion sections of this paper, we refer to this fly line as *cry^b^ Pdf^01^*. For the rescue studies using *pdfr-myc* in a *pdfr^5304^; ; cry^b^* mutant background, we generated recombinant strains of *pdfr-myc* and *cry^b^*, and crossed to *pdfr^5304^; ; cry^b^* flies. The recombinant flies were confirmed through single fly PCR and sequencing to check the possession of *pdfr-myc* transgene and *cry^b^* allele.

### Behavioral analyses

All locomotor activity experiments were conducted with 1–2 days-old male flies at 25°C using Trikinetics *Drosophila* activity monitor system. The light intensity for LD entrainment and LL was ∼300 lux, unless it is marked otherwise. All experiments were repeated two or more times with similar results, except for experiments that tested the effects of short and long days (Figure 3). For DD experiments, flies were entrained to 12∶12 hr LD cycles for 6 days and then released into DD for 9 days. For LL experiments, flies were kept for 5–6 days under 12∶12 hr LD cycles then for 9 days under LL. We used two different light meters (Field Scout (Spectrum Tecnologies (Plainfield, IL) and W/RS-232 (VWR Scientific)). The first produced measurements in micromoles/m^2^s and the second in lux. We tested the effects of three different light intensities: low (∼1–2 micromoles/m^2^s; ∼30 lux), a middle light (∼10 micromoles/m^2^s; ∼300 lux), and high (∼30 micromoles/m^2^s; ∼1,000 lux). We multiplied readings in micromoles/m^2^s by 0.342 to convert them to units of µW cm^−2^. Therefore, the middle and high light intensities we used are similar to those reported by Cusumano et al. [15] (300∼1,000 µW cm^−2^). To assess rhythmicity, we used χ2-periodogram analysis with 95% confidence cut-off as well as Signal-to-noise ratio (SNR) analysis [19]. We judged behavior during days 3–9 of DD or LL to be arrhythmic if the power was lower than 10 and width lower than 1, or if period fell outside the range, 18 to 30 hours. For phase analysis, we acquired acrophase values on the second day of LL cycles from individual flies using Clocklab software (Actimetrics, Evanston, IL), and then averaged those to acquire a group phase value for each condition.

### Immunocytochemistry

Immunocytochemistry was performed in wholemount using male flies. Fly brains were dissected in Ca^2+^-free fly saline and fixed for 60 min at RT in 4% (w/v) paraformaldehyde, 7% (v/v) picric acid in PBS (pH 7.4). After three washes with PBS-Tx (1× PBS with 0.3% triton-X100), dissected brains were incubated in 3% Normal Goat Serum in PBS-Tx for one hour at room temperature. The tissues were incubated in primary antibodies (dilutions below) for 48 hr at 4°C, and in 1∶1,000 diluted secondary antibodies for 24 hr at 4°C. Primary antibodies include mouse anti-MYC, rabbit anti-MYC, rat anti-PER, rabbit anti-βPDH, guinea pig anti-proPDF (anti-PAP), mouse anti-PDF, rabbit anti-CRY, and rabbit anti-ITP with dilution factors of 1∶1,000, 1∶400, 1∶500, 1∶2,000, 1∶1,000, 1∶100, 1∶1,000 and 1∶1,000, respectively [10], [17], [20]–[24]. Rabbit anti-MYC antibody and mouse anti-MYC antibody are commercially-available (Bethyl, Canada, and Cell Signaling, Danvers, MA, USA). For double staining with anti-CRY, 1∶200 dilutions of secondary antibodies were used. Secondary antibodies were conjugated to Alexa 488, Alexa 568, Cy3, or Alexa 633 (Molecular Probes, Eugene, OR; Jackson Immunoresearch, West Grove, PA).

Images from wholemount were obtained on an Olympus 500 Fluoview Confocal microscope and the lookup tables were used to set appropriate PMT voltage and laser power. Z-projection images represent stacks that contain 0.5 µm focal step images using 3-times Kalman averaging. Confocal stacks were projected in Image J and edited for contrast in Photoshop. For circadian time point images, different genotypes of flies at the same time points were treated in parallel for the whole procedure; identical confocal microscopy settings were applied to all genotypes and all time points. For quantification of PER levels and PER subcellular localization in the 5^th^ s-LNv, ITP(+) LNd, (Figure 6, Figure 10) and PDF(+) s-LNv (Figure 7), single focal plane images were obtained using 5-times Kalman averaging. Quantification was performed with Image J software. PER rhythm amplitudes were calculated by dividing the highest value with lowest value for each set. Lacking a suitable marker of sub-cellular domains for DN1, we simply counted the number that were PER(+) within 60 µm z-stacks from the posterior dorsal surface.
